# Innovative approaches towards an economic fusion reactor

**DOI:** 10.1093/nsr/nwz162

**Published:** 2019-10-30

**Authors:** Houyang Guo, Francis Y C Thio, Michl W Binderbauer, Richard J Buttery, Thomas R Jarboe, Rajesh Maingi, John S Sarff, Peter C Stangeby, Derek A Sutherland, Mickey R Wade, Michael C Zarnstorff

**Affiliations:** 1 General Atomics, USA; 2 Breakthrough Fusion International Corporation, USA; 3 School of Science, Xi’an Jiaotong University, China; 4 Tri Alpha Energy, USA; 5 Department of Aeronautics & Astronautic, University of Washington, USA; 6 Princeton University Plasma Physics Laboratory, USA; 7 Department of Physics, University of Wisconsin, USA; 8 Institute for Aerospace Studies, University of Toronto, Canada

Nuclear fusion potentially offers a clean, environmentally friendly and intrinsically safe energy source with an abundant fuel supply. Magnetic fusion energy research is approaching a new era of fusion power reactor design and cons-truction planning. New physics understanding and powerful predictive tools have become available for improving fusion performance, developing and optimizing various magnetic confinement concepts. Emerging transformative enabling technologies can potentially mitigate and transform some present physics challenges from the possibly insurmountable to the potentially solvable.

These recent innovations and advances in, as well as synergy between, *physics* and *technology* may increase absolute fusion performance at smaller scales, thus offering exciting opportunities to accelerate progress towards an economically viable fusion power reactor.

From nearly seven decades of worldwide effort to develop fusion energy, a vast knowledge base has been established of science and technology for creating and controlling high-temperature plasmas, ranging from relatively low-density magnetic-field confinement to extremely high-density laser-driven inertial confinement. The highest-performance approach towards steady-state fusion is a doughnut-shaped magnetic confinement configuration known as the tokamak, as evidenced by the joint international effort to build a power-plant-scale experiment, ITER (Latin for ‘the way’) [1]. ITER will achieve burning plasmas and access to actual reactor conditions. In particular, ITER will examine fusion alpha-particle effects on transport and stability that cannot be investigated in present-day experiments. Further, ITER will provide vital data for the licensing and engineering of a fusion reactor. The top priority of the present research is focused on resolving ITER modes of operation, including control and mitigation of transient and disruptive events driven by magnetohydrodynamic instabilities, and development of the operational scenarios and understanding needed to enable ITER to meet and potentially exceed its performance goals, while being compatible with the plasma–material interface to avoid damage to the walls. In addition, increased efforts are being made to develop innovations to address critical challenges for future fusion plants, including increased plasma stability, fusion reactor materials and plasma-facing components, tritium breeding and steady-state sustainment and heating technology. Many international participants have plans for prototype fusion power plants to follow ITER, such as the China fusion engineering test reactor (CFETR) [2] and the EU demonstration power plant [3].

**Figure 1.**
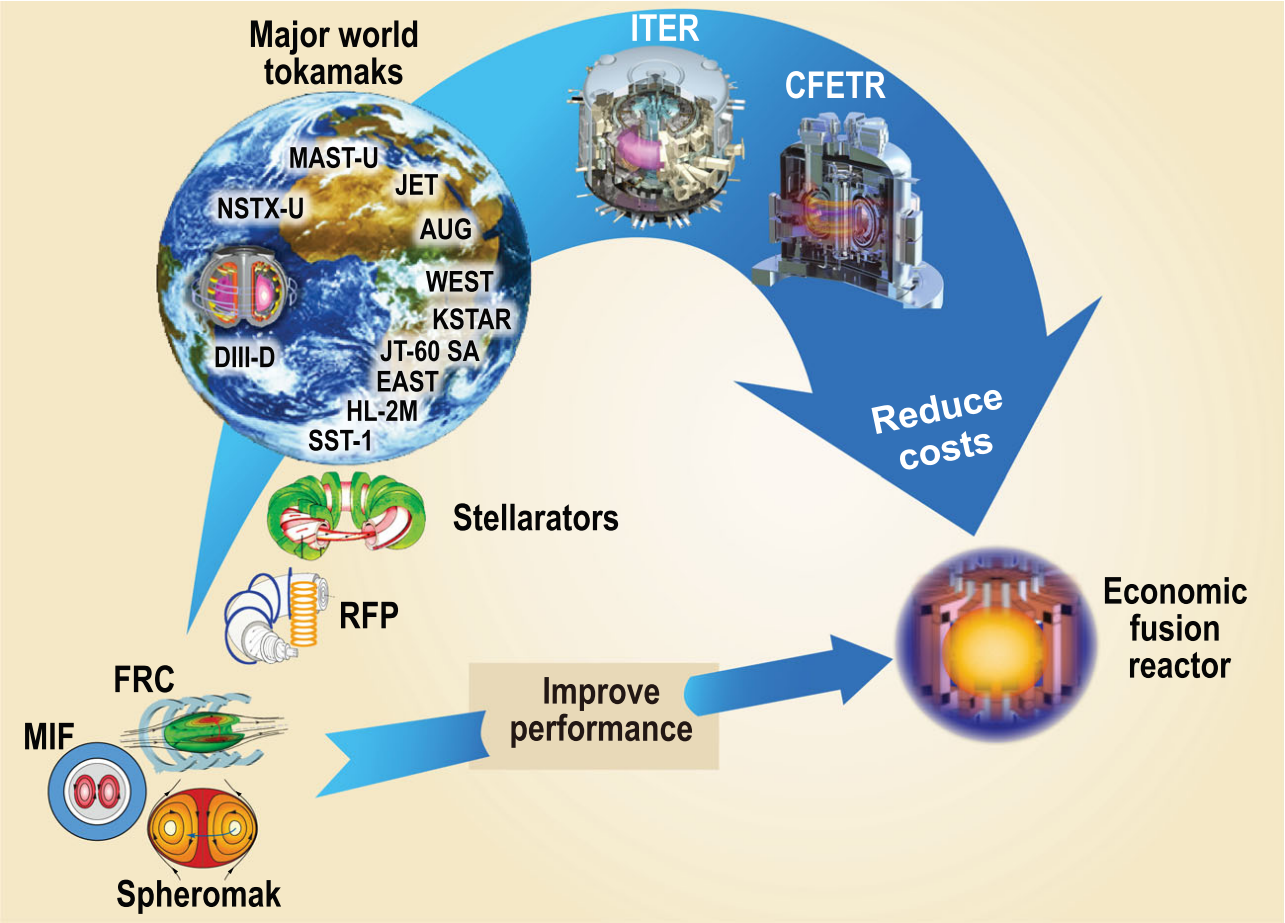
A potential dual-path approach towards an economic fusion reactor leveraging physics and engineering innovations: (1) Advanced magnetic confinement, relying on toroidal field magnet systems, including tokamak, stellarator and reversed field pinch (RFP), led by the tokamak; (2) Simplified magnetic topology, such as field reversed configuration (FRC) and spheromak, as well as magneto-inertial fusion (MIF), which are much less mature than the mainline tokamak approach; breakthroughs are needed to reach a similar technical readiness level to that for the advanced group.

Anticipating the scientific and technological success of ITER, demonstration of the economic viability, ease of maintenance and safety of fusion systems will then be at the forefront of next-step fusion energy efforts leading to ultimate commercial use. As indicated by Fig. 1, within the magnetic fusion effort this is pursued along two pathways towards an economic fusion reactor:

- Advanced magnetic confinement, relying on the toroidal field magnet system to increase the plasma stability, including the tokamak, stellarator and reversed field pinch (RFP) that has a smaller but substantial toroidal field;
- Simplified magnetic topology, which is a singly connected magnetic configuration having no toroidal field coils, such as the field reversed configuration (FRC) and the spheromak compact toroids, as well as hybridization of magnetic and inertial fusion (MIF).

The challenge of the first route is not least an engineering one—to reduce the cost for construction and operation, and to make the magnetic confinement system efficient, sustained and more compact. The major challenge today for the simpler magnetic configurations is more on the physics side, as they are much less mature and require significant improvements in their ability to confine high-temperature plasmas for the necessary durations relative to tokamaks or stellarators. Such a high-scientific-risk approach is currently largely driven by private enterprise, such as Tri Alpha Energy, Lockheed Martin and General Fusion, motivated by their highly desirable engineering features and potential for smaller and cheaper fusion power plants, if breakthroughs can be made in achieving fusion conditions.

On the physics front, the tokamak offers solutions in which good performance potentially aligns with configurations that are self-sustaining. Advances such as the recent discovery of the super-H mode of energy confinement in the DIII-D tokamak may enable still higher fusion performance [4], potentially lowering the capital cost of the corresponding power plant and moving economic fusion energy a step closer to reality. Energy confinement can be further improved by the use of lithium wall conditioning—by nearly five-fold as demonstrated in the National Spherical Tokamak Experiment (NSTX) [5]. A full tokamak reactor system analysis shows that very high confinement can help ease the challenge posed by the power exhaust, which is a critical issue for a compact fusion system, and can also reduce the required device size to sustain steady-state fusion power production [6,7]. As a major alternative magnetic fusion concept using nearly all external magnetic fields (both the main toroidal field and the poloidal field), the stellarator is inherently steady state without the need for current drive, and is free of disruptions. Optimization of the stellarator configuration shows promise in overcoming earlier major issues in neoclassical transport and improving fast particle confinement, achieving encouraging results including high temperatures ∼10 keV and record stellarator confinement times in the Wendelstein 7X experiment (W-7X) [8]. Advances in the understanding of the physics of magnetic reconnection and the ability to generate and control magnetic helicity are poised to spawn innovations in magnetic configurations suitable for creating fusion burning plasmas that are potentially substantially less expensive. Active control of the magnetic reconnection process in the MST RFP has been effective in making energy confinement in the RFP comparable to that in a tokamak when compared at low magnetic field [9]. Helicity injection current drive has been used to form and sustain the spheromak configuration fully inductively at low current [10]. This may provide a new pathway towards higher-performance sustained spheromaks. The axisymmetric form of oscillating helicity injection is also being developed for inductive sustainment of RFP plasmas [11]. The FRC appears to be extremely rugged, surviving highly dynamic translation and merging processes, and exhibiting a tendency for stable high beta (ratio of plasma to magnetic pressure) in a minimum energy state [12]. Confinement in the merged and neutral-beam sustained FRCs exhibits a significant improvement over conventional theta-pinch FRCs and a strong, favorable dependence on plasma temperature [13]. It is critical to demonstrate such favorable confinement scaling at high plasma temperatures for the next step in FRC development. The MIF in a pulsed mode combines a number of desirable features of magnetic and inertial fusion, especially with a stand-off driver like plasma jets [14]. All MIF embodiments can potentially avoid first-wall nuclear material issues by using thick flowing liquid wall, offering a potentially attractive approach towards producing burning plasma conditions. Note again that these *alternative* fusion concepts have highly desirable engineering features for a compact, economic fusion reactor, but have not yet been proven viable and so require further exploration.

On the technology front, a number of promising transformative enabling capabilities provide enormous opportunities to accelerate fusion science and technology towards power production [15], including artificial intelligence (AI) and machine learning, high critical-temperature superconductors (HTS), advanced materials and manufacturing, new RF current drive techniques, novel technologies in tritium fuel cycle control, as well as fast-flowing liquid metal plasma-facing components. In particular, AI and machine learning have been applied to optimize operation scenarios, improve control and strategies, and to mitigate or prevent disruptions. Recent advances in HTS such as REBCO could enable doubling of the magnetic field over present designs, leading to more than an order of magnitude increase in the fusion power density, reducing reactor core size and opening up the prospect of high field designs. Advanced manufacturing (3D printing) can revolutionize fusion material component design, and reduce the cost drivers, which has hampered stellarator construction to date. Flowing liquid metal plasma-facing components provide a self-healing surface and enable reactor-scale plasma exhaust in the presence of plasma and neutron fluxes that would damage solids, which is a major technical challenge for the development of a fusion plant.

In summary, a confluence of physics and technology innovations in recent years show great promise for accelerating fusion energy development in the next few decades and lowering its cost. For the mainline tokamak approach, innovations gained in new physics understanding may enable access to and sustainment of enhanced confinement beyond ITER baseline scenarios, which, coupled with high magnetic confinement fields, potentially allows for high fusion power density at reduced size and cost. Exciting progress has also been made in the stellarator approach, achieving high temperatures up to ∼10 keV. The physics is less mature for the other alternative fusion concepts, and breakthroughs are needed to reach a similar technical readiness level to that for tokamaks, in particular for those with simplified magnetic topology, as exemplified by compact toroids. A number of emergent enabling technological capabilities, heavily funded by major non-fusion applications, may help solve long-standing challenges for the development of a fusion power plant, such as fusion materials, power handling and tritium production.

## References

[1] ITER. http://www.iter.org (30 October 2019, date last accessed).

[2] Li J , Ni M, Lu Y. Natl Sci Rev 2019; 6: 382–3. 10.1093/nsr/nwz029 PMC8291618 34691881

[3] The demonstration power plant: DEMO. https://www.euro-fusion.org/programme/demo (30 October 2019, date last accessed).

[4] Snyder PB , Solomon WM, Burrell KH et al. Nucl Fusion 2015; 55: 083026.

[5] Maingi R , Kaye SM, Skinner CH et al. Phys Rev Lett 2011; 107: 145004. 22107204 10.1103/PhysRevLett.107.145004

[6] Wade MR . General Atomics Perspective on the US MFE Program in 2020s. Washington: Fusion Power Associates, 2018.

[7] Menard JE , Brown T, El-Guebaly L et al. Nucl Fusion 2016; 56: 106023.

[8] Klinger T , Andreeva T, Bozhenkov S et al. Nucl Fusion 2019; 59: 112004.

[9] Sarff JS , Almagri AF, Anderson JK et al. Nucl Fusion 2013; 53: 104017.

[10] Victor BS , Jarboe TR, Hansen CJ et al. Phys Plasmas 2014; 21: 082504.

[11] McCollam KJ , Blair AP, Prager SC *et al*. Phys Rev Lett 2006; 96: 035003. 16486717 10.1103/PhysRevLett.96.035003

[12] Guo HY , Binderbauer MW, Tajima T et al. Nat Commun 2015; 6: 6879. 25902924 10.1038/ncomms7897

[13] Binderbauer MW , Tajima T, Steinhauer LC et al. Phys Plasmas 2015; 22: 056110.

[14] Thio YCF , Hsu SC, Witherspoon FD et al. Fusion Sci Technol 2019; 75: 581–98.

[15] Maingi R , Lumsdaine A, Allain JP et al. Fusion Sci Technol 2019; 75: 167–77.

